# Spectrum of glomerular diseases causing acute kidney injury; 25 years experience from a single center

**DOI:** 10.12861/jrip.2015.24

**Published:** 2015-11-20

**Authors:** Rubina Naqvi, Muhammed Mubarak, Ejaz Ahmed, Fazal Akhtar, Sajid Bhatti, Anwar Naqvi, Adib Rizvi

**Affiliations:** ^1^Sindh Institute of Urology and Transplantation (SIUT), Karachi, Pakistan

**Keywords:** Acute kidney injury, Acute glomerulonephritis, Crescentic glomerulonephritis, Renal biopsy, Plasmapheresis

## Abstract

**Introduction:** Acute kidney injury (AKI) is common in nephro-urological practice. Its incidence, prevalence and etiology vary widely, mainly due to variations in the definitions of AKI.

**Objectives:** We aim to report the spectrum of glomerular diseases presenting as AKI at a kidney referral center in Pakistan.

**Patients and Methods:** An observational cohort of patients identified as having AKI which was defined according to RIFLE criteria, with normal size, non-obstructed kidneys on ultrasonography, along with active urine sediment, edema and new onset hypertension.

**Results:** From 1990 to 2014, 236 cases of AKI secondary to acute glomerulonephritis (AGN) registered at this institution. Mean age of patients was 27.94± 12.79 years and M:F ratio was 0.77:1. Thirty percent patients revealed crescents on renal biopsy. AGN without crescents was seen in 33.05% of cases. Postinfectious GN was found in 14.4%, lupus nephritis in 8.5% and mesangiocapillary GN in 3.4% cases. Renal replacement therapy (RRT) required in 75.84% patients. Pulse steroids were given in 45.33% cases followed by oral steroids. Pulse cyclophoshphamide was given in 23.7% cases and plasmapheresis was used in 3.38% cases. Complete recovery was seen in 44%, while 11.44% died during acute phase of illness. About 19.49 % developed chronic kidney disease (CKD) and 25.84% were lost to long- term follow-up.

**Conclusion:** Although glomerular diseases contribute only 4.19 % of total AKI at this center, morbidity associated with illness and its treatment is more marked than other AKI groups. Another notable factor is late referral of these patients to specialized centers resulting in undesirable outcome.

**Implication for health policy/practice/research/medical education:**

This study may guide the physicians other than nephrologist who should refer the patients with glomerular diseases to subject specialist at an early stage, so that may prevent young patients progressing into chronic kidney disease (CKD).

## Introduction

Acute kidney injury (AKI) is common in nephro-urological practice. Its incidence, prevalence and etiology vary widely, mainly due to variations in the definitions of AKI (1-5). It is worth emphasizing the point here that AKI is not a specific disease. Rather it represents a clinical manifestation of many medical and surgical diseases of the kidney (6). RIFLE criteria were formulated to standardize the nomenclature and categorization of AKI across the world (7).

An optimal management of AKI depends on the determination of underlying disease process (8). This often requires recourse to the invasive procedure of renal biopsy. An analysis of renal biopsy causes of AKI sheds considerable light on the prevailing pattern of renal diseases in a given area of the country (9). However, it must be kept in mind that the biopsy pattern does not truly reflect the prevailing pattern of renal diseases, as it is subject to significant bias with regard to the biopsy indications. These vary from center to center and from one nephrologist to the other (10). We have previously analyzed the spectrum of pathological lesions underlying acute renal failure (ARF) at our center. In that, the major cause of ARF was acute tubular necrosis, accounting for 38.6% of all cases. Acute glomerulonephritis (AGN) accounted for 31% of cases (11). AGN or rapidly progressive glomerulonephritis often presents with azotemia after short illness. A history of recent-onset hypertension, edema and active urine sediment are common clinical associations.

## Objectives

This study was carried out to determine the spectrum of glomerular causes of AKI in a tertiary care hospital for urological and nephrological diseases.

## Patients and Methods

A retrospective analysis of case records of patients presenting to nephrology service of Sindh Institute of Urology and Transplantation (SIUT) was carried out from January 1990 to December 2014. All patients of either sex who fulfilled the RIFLE criteria of AKI, with normal size, non-obstructed kidneys on ultrasonography, active urine sediment, edema and new onset hypertension, and in whom glomerular pathology was the prime cause of AKI, were included. Written informed consent was obtained from all patients prior to the performance of all procedures.

### Data

The case records of patients were scrutinized to retrieve following data items: age and sex of the patients, serum creatinine at presentation, relevant serology, renal biopsy findings, mode of renal replacement therapy (RRT) offered, pharmacological substances used in treatment and the final outcome of the disease.

### Pathological studies

At our center, two cores of native renal tissue are routinely obtained for full pathologic evaluation as described in detail in our previous paper (12). Briefly, one core is processed for light microscopy (LM) and is fixed in 10% buffered formalin. The other core is bisected into two pieces for electron microscopy (EM) and immunofluorescence (IF) study.

### Light microscopy

For LM, routinely 10 serial sections are cut and stained by hematoxylin and eosin (H&E), Masson’s trichrome stain, periodic acid Schiff (PAS), and silver (Gomori’s methenamine silver, GMS). In our laboratory renal tissue sections are cut at a thickness of 2 um for optimal evaluation of the microscopic details as reported previously (12).

### Immunofluorescence study

Tissue specimens for IF are snap-frozen and cut on Shandon Cryotome. The tissue is stained by the direct method using FITC-conjugated antisera mono-specific for IgG, IgA, IgM, C3 and C1q (Dako Inc., Glostrup, Denmark). The slides are visualized under the Epi-flourescense microscope and graded semiquantitatively as 0 to 4+ and distribution described as membranous or mesangial in a granular or liner pattern as described previously (12). IF findings on the biopsy specimens were obtained from the original renal biopsy reports.

### Electron microscopy

Tissue samples for EM are processed according to presently established technique (13). Briefly, EM tissue was fixed in 4% glutaraldehyde, postfixed in 1% osmium tetroxide at 0.02 M Sorenson phosphate buffer at pH 7.4, processed for electron microscopy and embedded in Eponate resin. Ultra-thin sections (100 nm) were cut on Leica ultramicrotome. Sections were stained on copper 300-mesh girds with Uranyl acetate and Lead citrate and examined with a JEM 1200 EX II electron microscope. EM was done on all biopsies with glomerular disease on LM.

### Ethical issues

The study protocol was in accordance with the Declaration of Helsinki. Informed consent was obtained and the issue of writing was discussed with institutional review committee and was approved.

### Statistical methods

The collected data was analyzed using IBM compatible SPSS for windows version 19.00 (SPSS Inc., Chicago, IL, USA). Simple descriptive statistics such as mean ± SD were used for variables such as age and clinical and laboratory features. Numbers (percentages) were used for categorical data.

## Results

**Figure 1:**
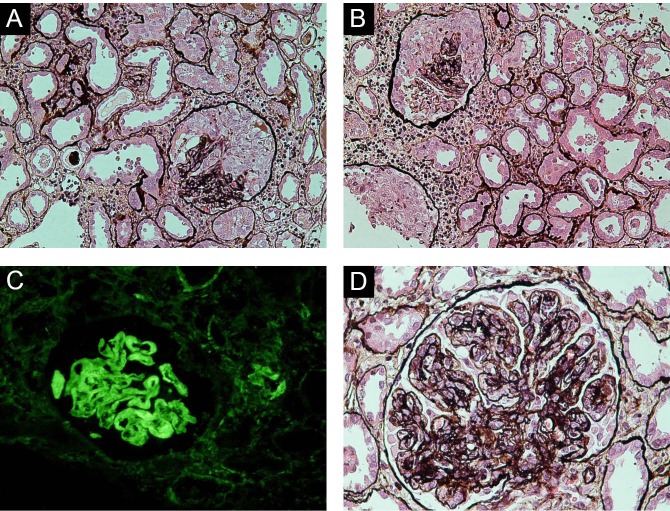
A) Medium-power view showing a soft, cellular crescent occupying the Bowman’s space in a case of crescentic GN. (silver stain, ×200). B) Medium-power view showing two glomeruli with soft, cellular crescents occupying the Bowman’s space in a case of crescentic GN. There is also interstitial inflammation. (silver stain, ×200). C) Anti-GBM. Immunofluorescence for IgG antibody showing bright, linear, ribbon-like positivity of IgG along the capillary walls. D) High-power view showing one glomerulus with diffuse marked mesangial proliferation, lobular attentuation, thickening of peripheral capillary walls and obliteration of capillary lumina in a case of membranoproliferative GN. (silver stain, ×200).

During the study period, 236 cases of AKI secondary to AGN were registered at this institution. Among these, 103 (43.6%) were males and 133 (56.4%) females and the male to female ratio was 0.77:1.The mean age of patients was 27.94 ± 12.79 years. The glomerular lesions underlying AKI are shown in Table 1. As is apparent from this table, upto 30% of patients revealed crescents on renal biopsies (Figures 1A -C). A representative picture of mesangiocapillary GN is shown in Figure 1D.

**Table 1:** Frequency of different causes (N = 236)

**Cause**	**No. of patients**	**%**
GN with Crescents	118	50
ANCA pos*	19	
Anti GBM pos*	8	
Lupus	16	
PIGN**	6	
GN without Crescents	78	33.5
Post Infectious GN	34 (6 with crescents)	14.40 (of total)
Lupus	20 (16 with crescents)	8.47 (of total)
Mesangiocapillary GN	8	3.38 (of total)

*Dual positivity in 2 cases. **PIGN = post infectious glomerulonephritis.

RRT was required in 75.84% of patients. Other treatment modalities given are shown in Table 2. Complete recovery was observed in 44% of patients, while, 11.44% died during acute phase of illness. A substantial number of patients (19.49%) developed chronic kidney disease (CKD) and 25.84% were lost to long-term follow-up (Table 3).

**Table 2:** Treatment modalities

**Treatment modality**	**No. of patients**	**%**
Renal replacement therapy	179	75.84
Pulse steroid followed by oral	107	45.33
Pulse cyclophosphamide	56	23.72
Plasmapheresis	8	3.38

**Table 3:** Outcome in different groups

**Cause**	**Complete recovery**	**Partial recovery**	**CKD**	**Expired**
GN without Crescents (n=78)	31	26	13	8
GN with Crescents (n=71)	30	18	19	4
Post Infectious GN (n=34)	23	7	2	2
Lupus nephritis (n=20)	9	2	4	5
Mesangiocapillary GN (n = 8)	6	1	1	0
Anti GBM (n = 8)*	0	3	3	2
ANCA positive (n=19)*	5	4	4	6

*Dual positivity in 2 cases

## Discussion

Various forms of AGN may present as AKI. Although, AGN as a cause of AKI in a particular case may be suspected on clinical grounds, it often requires recourse to the invasive procedure of renal biopsy, which currently represents the gold standard test for the diagnosis of medical conditions of the kidney. The frequency of AGN underlying AKI varies widely in different studies. This depends on many factors, including biopsy policies and biopsy indications at individual centers. Chugh and Sakhuja found AGN to be responsible for 9.8% of cases of AKI (13). In contrast, we found 31% cases of ARF due to different forms of GN in our previous study (11). The differences are most probably due to differences in biopsy indications, as we did perform biopsy in cases where history was not suggestive or where we had suspicion of ATN but recovery was delayed.

The spectrum of glomerulopathies presenting as AKI also varies depending on many factors, such as age, sex, race and region of the world, as well as the biopsy policies (11). Both crescentic and non-crescentic forms of AGN can present as AKI. The diagnosis of glomerular pathologies underlying AKI requires a high index of suspicion coupled with an algorithmic approach to laboratory investigations and renal biopsies.

Studies have shown that rapidly progressive glomerulonephritis (RPGN) can result from glomerular deposition of anti-GBM antibody, immune complexes, or from some as yet undefined mechanisms that does not involve glomerular antibody deposition. The later process may be cell mediated and resembles a small vessel vasculitis. Studies have shown patients having both ANCA and anti-GBM antibodies simultaneously positive, among these patients those with highest ANCA titers recovered renal function despite being initially on hemodialysis (14-18). In present series we had two patients with both antibodies positive.

Most cases of idiopathic RPGN are not accompanied by pathogenic glomerular immunoglobulin deposition and serological test also remain inconclusive (14).

## Conclusion

In conclusion, the results of this study show that AGN presents as AKI in a small but significant number of cases. An accurate diagnosis of these is essential for an optimal management of these patients. Delay in referral or diagnosis may result in unfavorable outcome in young patients.

## Limitations of the study

Our institution caters patient population from all over the country and many of the patients coming to us are from far distances, arrive in late stage of disease, so the treatment is not as successful as should be if initiated early.

## Authors’ contribution

RN: Data collection, design, literature search, statistical analysis, manuscript writing. MM: Helped in histopathological evaluation, description of methods and manuscript writing. EA: Helped with patients management and decisions towards management. FA: Helped with patients management and decisions towards management. SB: Helped with patients management and decisions towards management. AN: Dy Director of Institute, helped in provision of funds towards all steps of patient management. AR: Director of institute, helped in provision of funds towards all steps of patient management.

## Conflicts of interest

The authors declared no competing interests.

## Ethical considerations

Ethical issues (including plagiarism, data fabrication, double publication) have been completely observed by the authors.

## Funding/Support

None.
